# Opportunities for strengthening provider-initiated testing and counselling for HIV in Namibia

**DOI:** 10.1080/09540121.2015.1020281

**Published:** 2015-03-16

**Authors:** Tracy Davyduke, Ismelda Pietersen, David Lowrance, Selma Amwaama, Miriam Taegtmeyer

**Affiliations:** ^a^ Liverpool School of Tropical Medicine, Liverpool, UK; ^b^ National HIV/AIDS Control Program, Windhoek, Namibia; ^c^ Centers for Disease Control and Prevention, Windhoek, Namibia; ^d^ Ministry of Health and Social Services, Swakopmund State Hospital, Swakopmund, Namibia

**Keywords:** HIV testing, task shifting, sub-Saharan Africa, provider-initiated, Namibia, nursing

## Abstract

This short report identifies enablers and barriers to the uptake of provider-initiated testing and counselling for HIV (PITC) in Namibia and identifies key opportunities for strengthening this vital aspect of the national HIV response. We explored this through facility mapping, register reviews and qualitative methods including focus group discussions and in-depth interviews. Four health facilities (clinics and hospitals) in two regions were included in the study. We identified that PITC in Namibia was largely delivered by lay counsellors operating in designated rapid testing rooms located in health facilities and found a large number of missed opportunities for HIV testing through this model. Nurses did not see it as an integral part of their role, were not aware of HIV testing and counselling policy, felt inadequately trained and supported, and experienced staffing shortages. Institutional issues also acted as barriers to nurses performing or initiating discussions about PITC. Wider dissemination and implementation of policy, increasing privacy of consultation spaces and community sensitisation are simple measures that represent opportunities for strengthening this response and ensuring that symptomatic individuals who are unaware of their HIV status do not fall through the net.

## Introduction

Provider-initiated testing and counselling for HIV (PITC) shifts the onus for initiating HIV testing from the client to the health-care provider, thereby increasing the rates of detection of HIV infection and linkage to care in generalised epidemics (Kennedy et al., 2013; World Health Organization [WHO], 2007). The introduction of PITC in sub-Saharan Africa is a cost-effective (Walensky et al., 2011), acceptable and effective way of diagnosing HIV infection through a routine opt-out approach in antenatal care (ANC; Byamugisha et al., 2010), tuberculosis (TB; Odhiambo et al., 2008) and sexually transmitted infection (STI) clinics (Kharsany, Karim, & Karim, 2010). Where the offer of testing is not routine (such as in general outpatients or in-patient wards), uptake can be patchy with success dependant on local programmes, policies and staffing. PITC in these settings has been delivered largely through a task-shifting approach using either dedicated lay counsellors (Taegtmeyer et al., 2011), who require additional funding and are trained in a longer counselling protocol intended for the voluntary counselling and testing (VCT) approach; or nurses, many of whom have dual roles (Evans & Ndirangu, 2011).

Namibia has a mature, generalised HIV epidemic with an antenatal seroprevalence of 18.2% (Ministry of Health and Social Services [MoHSS], 2012), and over 50% of adults have never been tested for HIV (Ministry of Health and Social Services [MoHSS], 2008). The Ministry of Health and Social Services is guided by a National Strategic Framework (Ministry of Health and Social Services [MoHSS], 2010) and HIV testing and counselling (HTC) guidelines (Ministry of Health and Social Services [MoHSS], 2011) that outline PITC as an essential strategy for increasing testing and recognition of HIV. The HTC Guidelines recommend that PITC be conducted in room by the health-care worker who initiated the discussion. Thus, nurses and other health-care workers are expected to initiate and complete in-room testing and counselling. Community counsellors (CCs), who are lay counsellors co-located in health facilities, also take on this role, but their primary role is to see self-referrals direct from the community. Current practice is that whoever conducts pre-test counselling is expected to perform post-test counselling, but testing may be performed by a separate individual. PITC uptake in Namibia still remains low, and there is a need to increase nurse-led testing if goals of epidemic control are to be achieved. We set out to explore enablers and barriers to the uptake of PITC in the public sector in Namibia and identify opportunities to strengthen this service.

## Methods

Observational and qualitative methods were used (see Table 1). Data were collected over an eight-week period in 2012 at four public health facilities representing a cross section of facilities in Namibia: a clinic, a health centre, a district hospital and a referral hospital in the Khomas and Erongo regions.

**Table 1.** Methods of data collection.

Data collection method	Purpose	Sample
Client flow	To determine infrastructural barriers to PITC uptake	1 clinic, 1 intermediate referral hospital, 1 district hospital and 1 health centre
Register review	To document uptake of PITC after attending a facility for other reasons	All patient visits recorded in available patient registers on selected day, *n* = 728
FGDs	To explore provider perceptions of PITC delivery	5 FGDs – nurses from all sites, *n* = 29: 12 registered, 17 enrolled (1 male; 2 nurses participated in both IDIs and FGDs)
IDIs	To explore client flow and provider perceptions of PITC	14 nurses: 9 registered, 5 enrolled (2 males); 4 CCs (1 male)
KIIs	To understand policy-maker perceptions of PITC	Programme managers and quality assurance officers within the MoHSS, HIV programme donors, implementing partners, and training and laboratory institutions, *n* = 11 (5 males)

Note: All data collection was completed by T. Davyduke; topic guides were used for all interviews/discussions.

FGD, focus group discussion; IDI, in-depth interview; and KII, key informant interview.

We performed a comparative analysis of client flow, facility infrastructure and process across sites, assessing distance to counselling and testing rooms, designated processes and wait times. Patient registers were compared with HTC records to determine missed opportunities for testing. The qualitative data were analysed using a framework approach with a clear study design, independent review of transcripts and external validation through feedback to key stakeholders to ensure trustworthiness.

## Findings

The factors that determine PITC uptake arose from three emerging themes, reflecting the perspectives of our participants, the concomitant register reviews and facility observations. The preference of busy clinical staff for referral to designated CCs led to a range of missed opportunities for PITC. Human resource management and heavy workloads as well as infrastructural barriers and limited awareness of policy emerged as important themes from the qualitative data.

### Preference for referral leads to missed opportunities

**Figure 1.**
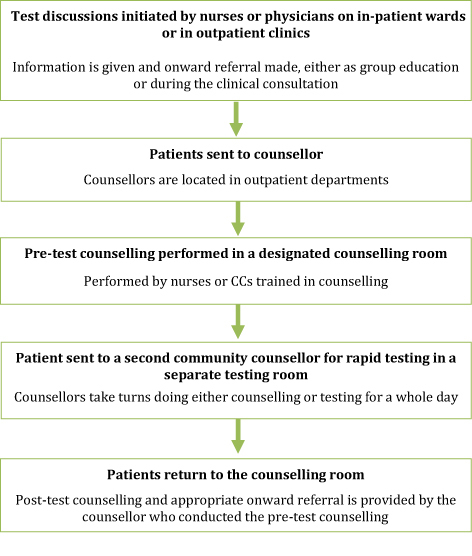
PITC steps.

Observational data, register reviews and interviews all showed that co-located CCs performed all of the HIV testing reported in the time period of this study. Counselling and testing was performed in a designated and physically separate room, utilising a full VCT protocol rather than the abbreviated PITC protocol (see Figure 1 for a description of the observed HTC process and client flow). The provision of HTC was during the CC's working hours only:

Umm … ok there are counsellors right now. So for me, I don't see the need to counsel a patient if there is a counsellor who is going to do the counselling AND the testing. (FGD, Registered Nurse, PHC)

During the period of observation, the majority of facility visits did not result in a testing episode: 728 in-patient and outpatient encounters resulted in 50 HIV tests. Few nurses outside of TB and ANC departments indicated that they routinely initiated HIV testing discussions or referral, and there were no reports of nurses performing in-room testing. ANC and TB clinics performed well with 42 out of 44 new ANC patients with negative or unknown status (95%) being sent for HTC. Of the new TB patients seen, 5/10 (50%) had HIV results recorded in their TB patient file and 5/10 (50%) were listed as unknown status.

### Human resource management

Of the 41 nurses interviewed, 11 were trained in counselling, 11 in rapid testing and only 5 in both. Despite training, interviews revealed that nurses did not see PITC as an integral part of their current role as they were not aware of HTC policy and felt inadequately trained and supported. Support systems were not seen as institutionalised, and nurses trained in counselling, as well as key informants from training institutions, expressed a lack of time for follow-up and support of nurses after training. All types of interviewees reported nursing shortages as a major barrier to initiating testing discussions or providing PITC:

Yeah [shortage of staff] is the big issue, like I said we are four … so if I have to screen my patient and counsel and test I don't know how many patients I will see for the morning. (IDI, Registered Nurse, PHC)

### Policy dissemination and infrastructural barriers

Interviewees reported that it was easier to initiate HTC and explain the rationale for testing in TB, ANC and STI settings where there were clear policies in place for routine testing, and patients were sensitised to being asked to test for HIV:

They are not coming with that frame of mind to that facility. Somebody that goes because they are worried about their own risks, issues or, even you know, even pregnant women already know it's part of the package. But the rest of the community, I think we need to inform them. (Key Informant, MoHSS)

National HTC Guidelines clearly outlined PITC as a standard component of medical care, but at the time of data collection, few nurses were aware of these:

Our guidelines were not very clear. Although PMTCT was there on board but the guidelines was not really coming forth to say healthcare workers can fully give information or go on about [PITC] … (Key Informant, MoHSS)

Nurses felt policies would help them overcome HIV stigma as it gave them an easier route into discussion of HIV, avoiding moral judgements. However, they cited a lack of privacy as a barrier in a context that still stigmatised HIV. While the designated rooms for counselling and rapid testing were sometimes marked with a potentially stigmatising biohazardous waste sign, these were at least private compared to outpatient consultations, which were conducted in shared spaces and felt by participants to be unsuitable for such personal discussions:

… you are sitting 2 in that room and sometimes the privacy is not so conducive so it is also difficult to pop out with “don't you think you should go for HIV test?” … the other patient that is listening is like hmmm. (FGD, Registered Nurse, PHC)

## Discussion

Our findings indicate that there are opportunities at all levels to strengthen PITC in Namibia, and many of these are being addressed in the recently revitalised National Strategy for HTC (Ministry of Health and Social Services [MoHSS], 2014). Despite significant progress with PITC in ANC, TB and STI clinics in Namibia, where PITC can be considered a duty of care to patients, the offer and conduct of testing for general outpatients and hospital in-patients lags behind. Nurses in Namibia have de-linked initiating and providing HTC, taking ownership of initiating HTC discussions in some settings but not seeing testing and counselling as part of their current role. A good PITC programme is “low hanging fruit” as far as HTC programming is concerned and should be a priority in high prevalence settings if epidemic control is to be achieved through identification and linkage to care and treatment of newly diagnosed individuals. Additionally, PITC is important for individual benefit as well as public health gain and acts as a safety net for those who have not previously tested through other routes (Topp et al., 2011).

Infrastructural and institutional issues may be relatively simple to address. The lack of dissemination of guidelines has been reported as an operational challenge in other settings (Roura, Watson-Jones, Kahawita, Ferguson, & Ross, 2013). The physical separation of testing areas from the point of clinical contact in Namibia presents an opportunity for patient dropout. Settings where HTC is offered on-site and at the point-of-care, such TB and ANC have shown increased uptake (Topp et al., 2011) and acceptability of testing (Dalal et al., 2011). A multicentre trial evaluating models of PITC that include referral to on-site HTC after clinical consultation, HTC provided during the clinical consultation by the health-care worker and HTC provided to patients before their clinical consultation by a health-care worker or lay counsellor is currently under way (Mc Naghten et al.,^2012^).

In line with WHO recommendations (WHO, 2007) and findings on community barriers to PITC (Musheke et al., 2013), community sensitisation was seen as essential by our participants. HTC was more routinely offered in ANC and TB settings, where patients were expecting it. Studies in Kenya on the use of mass media campaigns and community sensitisation for promotion of HTC have shown increased testing uptake (Marum, Morgan, Hightower, Ngare, & Taegtmeyer, 2008; Odhiambo et al., 2008), and dissemination of policy and changes in practice at facilities can quickly spread through community networks.

Chronic human resource shortages are however harder to address. Adding routine PITC is perceived as increasing workload (Pope et al., 2010), and nurses often feel there is not enough time to provide PITC in environments where they are already short staffed. The lack of mentoring and supervision nurses face when implementing PITC can undermine the success of PITC programmes and threaten the quality of service (Evans & Ndirangu, 2011). In response, task shifting to lay counsellors has been shown to be acceptable (Sanjana et al., 2009), increase programme efficiency, access and affordability (Callaghan, Ford, & Schneider, 2010), and reduce workload for nurses (Sanjana et al., 2009). This is only part of the solution however, and as the Namibian setting is heavily reliant on CCs, the success of the PITC programme is undermined by low rates of referral to the CCs, restricted opening times and poor linkages between services. This necessitates a two-pronged approach that includes PITC proactively initiated and, in some cases, provided by health-care workers.
